# Effectiveness and safety of lenvatinib plus anti‐programmed death‐1 antibodies in patients with hepatocellular carcinoma: A real‐world cohort study

**DOI:** 10.1002/cam4.5661

**Published:** 2023-02-15

**Authors:** Ming‐Hao Xu, Cheng Huang, Mei‐Ling Li, Xiao‐Dong Zhu, Chang‐Jun Tan, Jian Zhou, Jia Fan, Hui‐Chuan Sun, Ying‐Hao Shen

**Affiliations:** ^1^ Department of Liver Surgery, Zhongshan Hospital Fudan University Shanghai China

**Keywords:** ALBI grade, anti‐PD‐1 antibody, Child‐Pugh class, hepatocellular carcinoma, lenvatinib

## Abstract

**Objective:**

Lenvatinib plus anti‐programmed death‐1 (anti‐PD‐1) antibody combinations have shown potent anti‐tumor effect in phase I/II trials in advanced or unresectable hepatocellular carcinoma (HCC), but real‐world data are limited.

**Methods:**

To investigate the effectiveness and safety of lenvatinib plus anti‐PD‐1 antibodies in a real‐world cohort, we retrospectively evaluated 210 patients with unresectable or advanced HCC treated with these regimens between October 2018 and February 2022.

**Results:**

The objective response rate and disease control rate per Response Evaluation Criteria in Solid Tumors (RECIST) v1.1 were 28.1% and 75.2%. Median overall survival (OS) and progression‐free survival (PFS) in the overall cohort were 17.2 and 8.4 months, respectively. Median OS and PFS of patients receiving first‐line treatment reached 18.9 and 9.6 months. Median OS was significantly longer in patients with Child‐Pugh class A versus B (18.8 vs. 5.9 months, respectively), as was median PFS (9.1 vs. 4.4 months). Patients with albumin–bilirubin (ALBI) grade 1 versus grade 2/3 also had significantly greater median OS (23.5 vs. 13.4 months). Treatment‐related adverse events (AEs) occurred in 79.5% of patients. Patients with ALBI grade 2/3 had a higher rate of grade 3/4 AEs than patients with ALBI grade 1 (57.5% vs. 38.5%).

**Conclusion:**

Lenvatinib combined with anti‐PD‐1 antibody therapy was effective in patients with sufficient liver function reserve. Further study is needed to improve therapeutic efficacy and AE management in patients with Child‐Pugh class B or ALBI grade 2/3.

## Graphical abstract

The present study evaluated a real‐life cohort of 210 patients with unresectable or advanced hepatocellular carcinoma who received treatment with lenvatinib plus anti‐programmed death‐1 antibodies between October 2018 and March 2022. The results are encouraging, with median overall survival of 17.2 months and objective response rate of 28.1%.

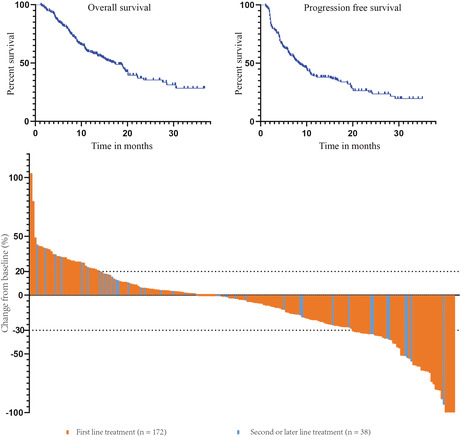

## 1 INTRODUCTION

Liver cancer is the fourth leading cause of cancer‐related mortality worldwide,^1^ among which hepatocellular carcinoma (HCC) accounts for more than 90% of cases.^2^ By the time of diagnosis, most patients have advanced disease, which has a poor prognosis and is not generally amenable to curative local therapies.^3^ Systemic options for first‐line treatment of advanced HCC include the targeted therapies sorafenib, which has been available for more than a decade,^4^ and lenvatinib, which was approved relatively recently.^5^ For sorafenib‐experienced patients, the anti‐programmed death‐1 (anti‐PD‐1) antibody immune checkpoint blockers (ICBs) nivolumab and pembrolizumab have been granted accelerated approval in the US.^6^, ^7^ In addition, ICBs have been investigated in combination with targeted therapies, and bevacizumab plus atezolizumab were recently approved for first‐line treatment of advanced HCC.^8^

Among other combinations of targeted therapies with ICBs that have been evaluated, lenvatinib plus various anti‐PD‐1 antibodies have shown potent anti‐tumor efficacy in early phase clinical studies. For example, lenvatinib plus pembrolizumab provided an objective response rate (ORR) of 36% in the phase Ib KEYNOTE‐524 trial,^9^ while lenvatinib plus nivolumab provided an ORR of 76.7% in the Phase Ib Study 117.^10^ Promising activity has also been reported for camrelizumab plus apatinib, with ORRs of 34.3% and 22.5%, respectively, in the first‐ and second‐line treatment cohorts of the RESCUE trial.^11^ Based on the encouraging data from phase I/II studies, there is considerable interest in the development of novel combination regimens, especially lenvatinib plus anti‐PD‐1 antibody combinations. Although the recent phase III randomized trial of lenvatinib plus pembrolizumab versus lenvatinib in patients with advanced or unresectable HCC failed to reach the primary endpoint, lenvatinib plus pembrolizumab combinations reached a first‐line treatment overall survival (OS) of 21.2 months, which is the best performance so far.^12^

Lenvatinib may reverse the immunosuppressive function of vascular endothelial growth factor in the tumor microenvironment, thereby improving the efficacy of anti‐PD‐1 antibodies.^13^ Due to the lack of overlap in the main adverse events (AEs) of targeted agents and ICBs, combination therapy is not expected to exacerbate toxicity compared with the respective monotherapy regimens.^14^ However, the LEAP‐002 trial suggested that combination of anti‐PD‐1 antibodies may increase the probability of AEs without significantly increasing OS.^12^ In real‐world practice, patients receiving combination therapy often suffer from poor liver function, due to the high tumor load and/or adverse effects of prior treatment. Based on either Child‐Pugh class or albumin–bilirubin (ALBI) score, impaired liver function has been associated with poor OS in previous trials, whether evaluating targeted therapies or ICBs.^15^, ^16^, ^17^ There is an outstanding need for a large dataset evaluating treatment outcomes and toxicity profiles in real‐world patients, including those with poor liver function, especially when treated with combinations of targeted therapies and ICBs. Here, we report the real‐world data on the efficacy and safety of lenvatinib plus anti‐PD‐1 antibodies in a retrospective cohort of patients with unresectable or advanced HCC.

## 2 PATIENTS AND METHODS

### 2.1 Study population

This retrospective, unpaired, single‐center study cohort comprised 224 patients treated with lenvatinib plus anti‐PD‐1 antibodies in combination for unresectable or advanced HCC at our medical center since the study began on October 2018, and cutoff was set at February 2022. HCC was diagnosed by typical images of contrast‐enhanced magnetic resonance imaging (MRI) or computed tomography (CT), according to the guidelines for the Diagnosis and Treatment of Hepatocellular Carcinoma (2019 Edition).^18^ Patients received combination therapy, rather than locoregional treatment, due to advanced stage HCC, insufficient future liver volume after resection (<40% of standard liver volume in patients with liver cirrhosis, or <30% of standard liver volume in patients without liver cirrhosis) or being beyond the up‐to‐seven criteria.^19^ After excluding patients who did not complete at least one cycle of combination therapy and assessment (*n* = 14), 210 patients were ultimately included in the analysis. Detailed baseline characteristics of the enrolled patients are available in Table 1. The 14 patients failed to complete one cycle of treatment and assessment due to refusal to further treatment or follow‐up loss. The study was approved by the Zhongshan Hospital Research Ethics Committee. Written informed consent was signed by patients before combination treatment.

**TABLE 1:** Baseline characteristics.

Variable^a^	Overall (*n* = 210)	According to ALBI grade		
		Grade 1 (*n* = 104)	Grade 2/3 (*n* = 106)	*p* value
Age, years	57 (24–83)	53 (25–83)	57 (24–76)	0.134
Sex				
Male	188 (89.5)	94 (90.4)	94 (88.7)	0.687
Female	22 (10.5)	10 (9.6)	12 (11.3)	
ECOG PS				
0–1	200 (95.2)	102 (98.1)	98 (92.5)	0.050
2	10 (4.8)	2 (1.9)	8 (7.5)	
HBsAg				
Positive	168 (80.0)	82 (78.8)	86 (81.1)	0.679
Negative	42 (20.0)	22 (21.2)	20 (18.9)	
BCLC stage				
A/B	52 (24.8)	35 (33.7)	17 (16.0)	0.003
C	158 (75.2)	69 (66.3)	89 (84.0)	
Child–Pugh class				
A	195 (92.9)	104 (100)	91 (85.8)	<0.001
B	15 (7.1)	0	15 (14.2)	
Extrahepatic metastasis				
None	129 (61.4)	68 (65.4)	61 (57.5)	0.243
Bone	11 (5.2)	6 (5.8)	5 (4.7)	
Lung	32 (15.2)	14 (13.5)	18 (17.0)	
Lymph node	24 (11.4)	13 (12.5)	11 (10.4)	
Abdominal cavity/peritoneum	18 (8.6)	11 (10.6)	7 (6.6)	
Macrovascular invasion				
No	109 (51.9)	63 (60.6)	46 (43.4)	0.013
Yes	101 (48.1)	41 (39.4)	60 (56.6)	
Treatment line				
First	172 (81.9)	85 (81.7)	87 (82.1)	0.948
Second or later	38 (18.1)	19 (18.3)	19 (17.9)	
AFP				
>400 ng/mL	107 (51.0)	47 (45.2)	60 (56.6)	0.098
≤400 ng/mL	103 (49.0)	57 (54.8)	46 (43.4)	
Albumin, g/L	40.0 (26–52)	43.0 (38–52)	36.0 (26–42)	<0.001
Total bilirubin, μmol/L	16.2 (3.8–116)	13.2 (4.8–28.6)	19.3 (3.8–115)	<0.001
PIVKA‐II, mAU/mL	3762 (12–75,000)	1502.5 (12–75,000)	8231.5 (17–75,000)	0.006
Maximum tumor diameter, cm	10.5 (0.6–23.3)	8.8 (0.6–23.3)	12.9 (1.3–31.9)	<0.001

Abbreviations: AFP, α‐fetoprotein; ALBI, albumin‐bilirubin; BCLC, Barcelona Clinic Liver Cancer; CA 19‐9, carbohydrate antigen 19‐9; CEA, carcinoembryonic antigen; ECOG PS, Eastern Cooperative Oncology Group performance status; HBsAg, hepatitis B surface antigen; PIVKA‐II, protein induced by vitamin K absence or antagonist‐II.

^a^ Categorical variables are summarized as *n* (%). Continuous variables are summarized as median (range).

### 2.2 Treatment

Patients weighed <60 kg or ≥60 kg received lenvatinib 8 or 12 mg/day, respectively, combined with one of the following anti‐PD‐1 antibody regimens: nivolumab 3 mg/kg or camrelizumab 200 mg every 2 weeks; or pembrolizumab 200 mg, sintilimab 200 mg, tislelizumab 200 mg; or toripalimab 280 mg every 3 weeks (Table S1). As no anti‐PD‐1 antibodies were approved for first‐line treatment of advanced HCC during the study period, anti‐PD‐1 antibody selection in this setting was principally based on treatment cost and available clinical data, including clinical trials of anti‐PD‐1 agents,^20^, ^21^ as well as our previous study suggesting comparable efficacy of different anti‐PD‐1 antibodies as monotherapy (ORR of 15%–20%)^7^, ^20^, ^21^ and combination therapy (ORR of 34%–36%).^7^, ^11^, ^22^ Combination treatment was continued until emergence of intolerable AEs or progressive disease (PD). During the treatment period, patients (the majority belonged to partial response [PR]) eligible for downstaging surgical resection underwent surgical treatment, and relevant details are demonstrated in our previous research.^23^, ^24^ Patients with PD received other drug regimens, locoregional treatment including tanscatheter arterial chemoembolization, or symptomatic treatment as appropriate.

### 2.3 Assessments

Tumor response was evaluated by abdominal contrast‐enhanced MRI or CT every 2 months according to Response Evaluation Criteria in Solid Tumors (RECIST) v1.1.^25^ AEs were categorized and graded according to the Common Terminology Criteria for Adverse Events (CTCAEs) v5.0. OS was defined as the time from beginning of combination treatment to death or censoring at the date of last follow‐up. PFS was defined as the time from initiation of combination treatment to disease progression or death.

### 2.4 Statistics

Continuous variables were shown as medians (range) and compared by Mann–Whitney *U*‐test or Student's *t*‐test. Categorical variables were compared using Fisher's exact or chi‐squared tests, as appropriate. OS and PFS curves were plotted using the Kaplan–Meier method. Multivariate Cox proportional hazards regression was performed in a stepwise manner using variables with *p* values of <0.05 during univariate analysis. *p* values of <0.05 were regarded as statistically significance. Statistical analyses were conducted in SPSS and R software.

## 3 RESULTS

### 3.1 Baseline characteristics

Baseline characteristics of the 210 patients included are presented in Table 1. One hundred and seventy‐two patients (81.9%) received combination therapy as first‐line treatment. Of the 38 patients who received non‐first‐line treatment, 35 received combination therapy as second‐line treatment, with the details of prior treatments shown in Table S2, and three patients received combination therapy as third‐line treatment, while receiving prior treatment of sorafenib and regorafenib beforehand. Nine (4.3%), 43 (20.5%), and 158 (75.2%) patients had Barcelona Clinic Liver Cancer (BCLC) stage A, B, and C disease. A total of 195 patients (92.9%) had Child‐Pugh class A, and 15 patients (7.1%) had Child‐Pugh class B. ALBI grade 1 was reported in 104 patients (49.5%), grade 2 in 106 patients (50.0%), and grade 3 in 1 patient (0.5%). Compared with patients with ALBI grade 1, those with ALBI grade 2/3 had a significantly higher prevalence of ECOG PS 2 (*p* = 0.050), BCLC stage C (*p* = 0.003), Child‐Pugh class B (*p* < 0.001), and macrovascular invasion (*p* = 0.013). Moreover, patients with ALBI grade 2/3 had higher baseline total bilirubin (*p* < 0.001), protein induced by vitamin K absence or antagonist‐II (PIVKA‐II) (*p* = 0.006), and maximum tumor diameter (*p* < 0.001), and lower albumin (*p* < 0.001) than patients with ALBI grade 1.

### 3.2 Treatment outcomes

**FIGURE 1:**
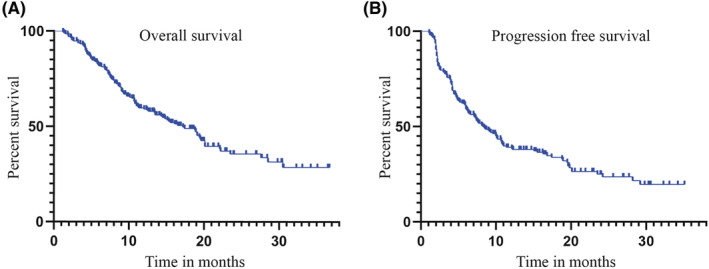
Kaplan‐Meier analyses of overall survival (A) and progression free survival (B) in the overall cohort.

**FIGURE 2:**
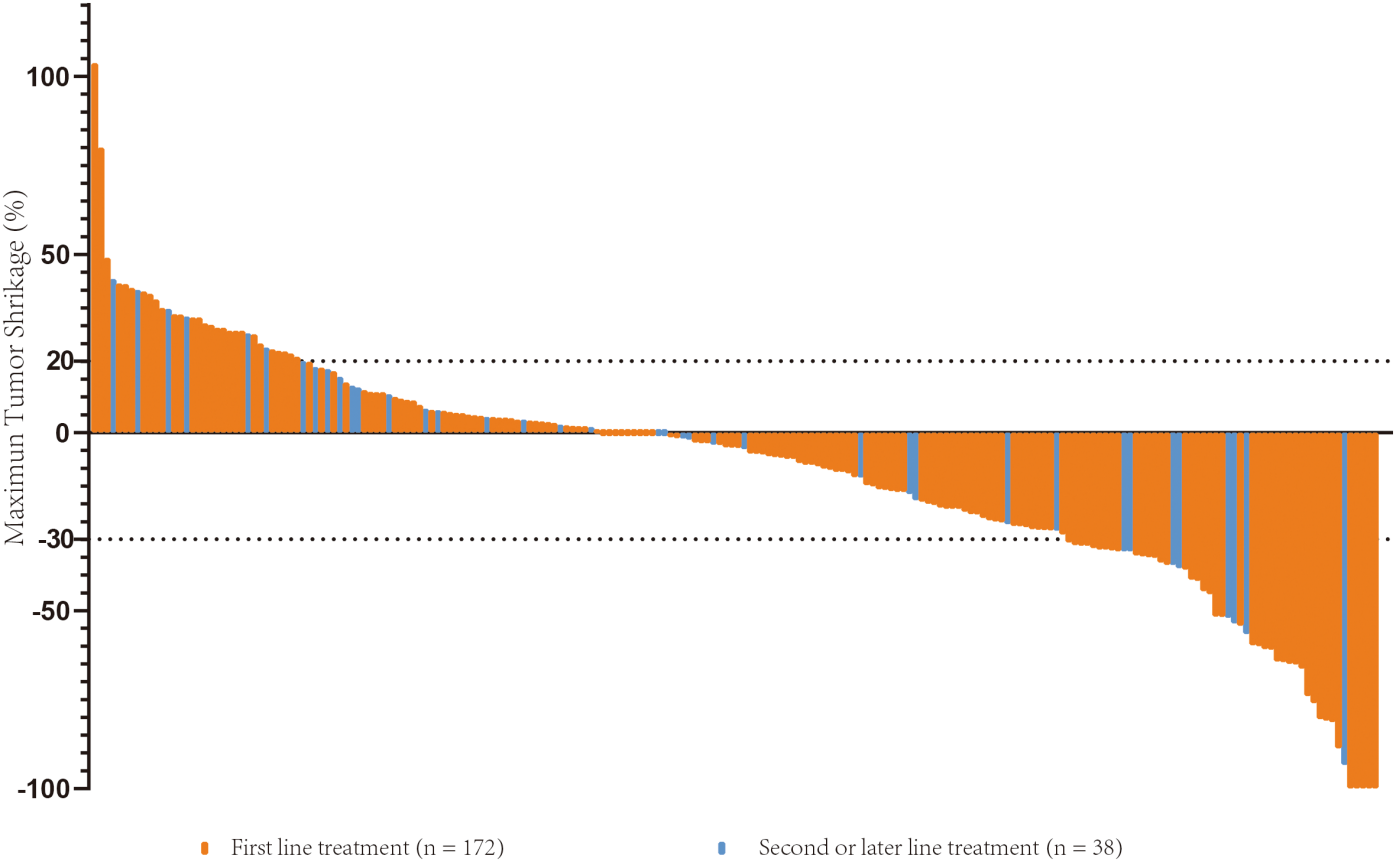
Waterfall plot of changes in tumor maximum diameter in the overall cohort by combination therapy of lenvatinib plus anti‐programmed death‐1 antibodies. The target areas of tumors were examined in each patient and tumor response was assessed by Response Evaluation Criteria in Solid Tumors (RECIST) v1.1.

Median follow‐up was 19.8 months (interquartile range, 13.7–27.6 months), and the maximum duration of follow‐up was 36.7 months. At the time of the analysis, 112 patients died, and median OS was 17.2 months (95% confidence interval [CI], 13.8–20.6 months) (Figure 1A). A total of 141 patients had disease progression events recorded, and median PFS was 8.4 months (95% CI, 6.6–10.2 months) (Figure 1B). The best response was complete response in 5 patients (2.4%), PR in 54 patients (25.7%), stable disease in 99 (47.1%) patients, and PD in 52 patients (24.8%) (Figure 2). Therefore, the ORR per RECIST v1.1 was 28.1% and disease control rate (DCR) was 75.2%. The ORR or DCR did not differ significantly according to ALBI grade (*p* = 0.946, *p* = 0.230), Child‐Pugh class (*p* = 1.000, *p* = 0.059), or line of therapy (*p* = 0.286, *p* = 0.136) (Table 2).

**TABLE 2:** Tumor response according to RECIST v1.1.

Best response	Overall	ALBI grade			Child‐Pugh class			Line of therapy		
		1	2/3	*p* value	A	B	*p* value	First	Second or later	*p* value
	(*n* = 210)	(*n* = 104)	(*n* = 106)		(*n* = 195)	(*n* = 15)		(*n* = 172)	(*n* = 38)	
CR	5 (2.4)	5 (4.8)	0 (0)		5 (2.6)	0 (0)		5 (2.9)	0 (0)	
PR	54 (25.7)	24 (23.1)	30 (28.3)		50 (25.6)	4 (26.7)		46 (26.7)	8 (21.1)	
SD	99 (47.1)	53 (51.0)	46 (43.4)		95 (48.7)	4 (26.7)		82 (47.7)	17 (44.7)	
PD	52 (24.8)	22 (21.2)	30 (28.3)		45 (23.1)	7 (46.7)		39 (22.7)	13 (34.2)	
ORR	59 (28.1)	29 (27.9)	30 (28.3)	0.946	55 (28.2)	4 (26.7)	1.000	51 (29.7)	8 (21.1)	0.286
DCR	158 (75.2)	82 (78.8)	76 (71.7)	0.230	150 (76.9)	8 (53.3)	0.059	133 (77.3)	25 (65.8)	0.136

*Note*: Data are presented as *n* (%).

Abbreviations: ALBI, albumin‐bilirubin; CR, complete response; PD, progressive disease; PR, partial response; RECIST, Response Evaluation Criteria in Solid Tumors; ORR, objective response rate; SD, stable disease.

**FIGURE 3:**
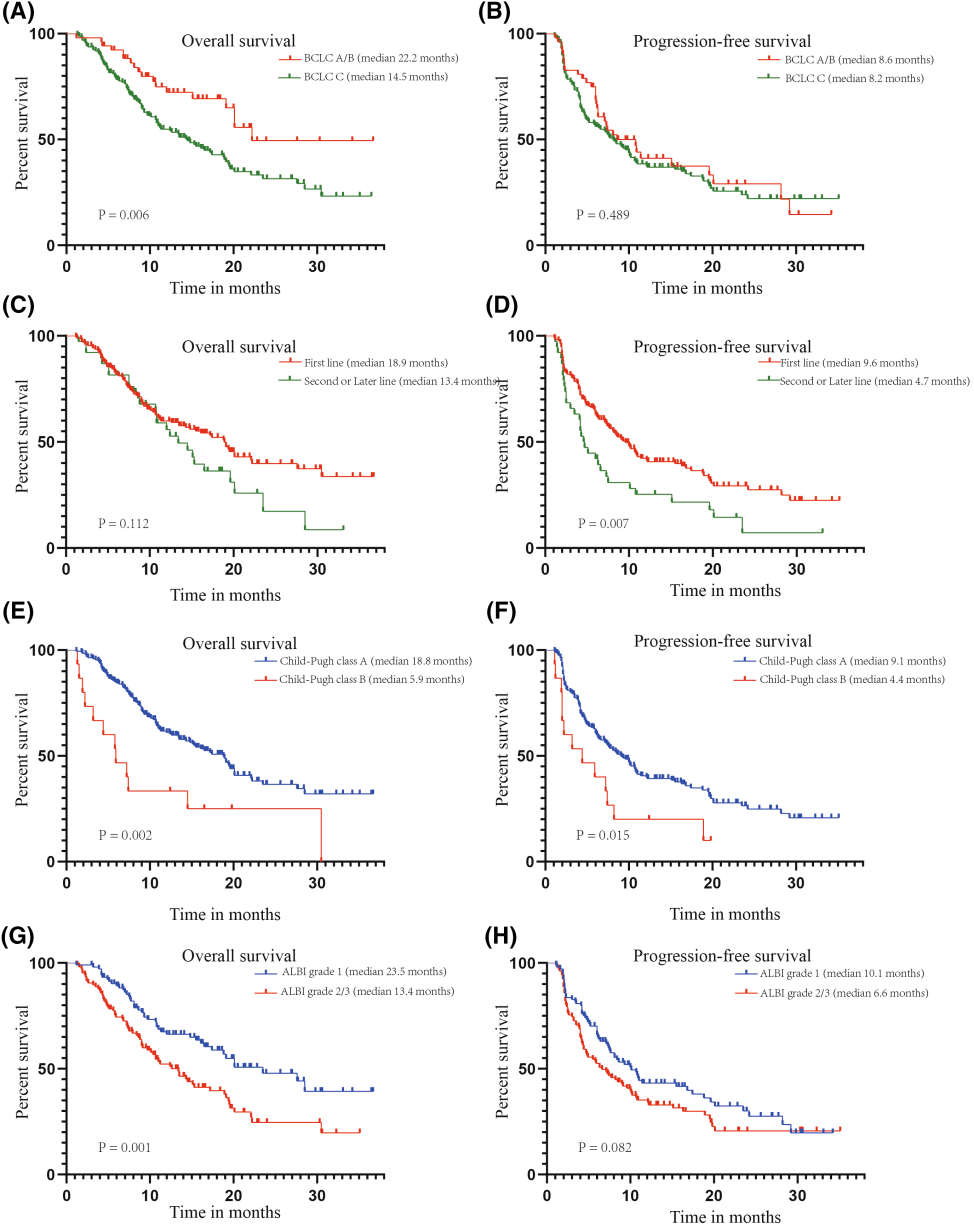
Overall survival (A, C, E, G) and progression free survival (B, D, F, H) according to BCLC stage (A, B), treatment line (C, D), Child‐Pugh class (E, F), and ALBI grade (G, H) in the overall cohort. ALBI, albumin–bilirubin; BCLC, Barcelona Clinic Liver Cancer.

Patients with BCLC stage A/B had significantly longer OS than those with BCLC stage C (medians, 22.2 vs. 14.5 months; hazard ratio [HR], 0.558; 95% CI, 0.368–0.846; *p* = 0.006) (Figure 3A). In contrast, no significant difference in PFS was found between BCLC stages A/B and C (medians, 8.6 vs. 8.2 months; HR, 0.875; 95% CI, 0.598–1.278; *p* = 0.489) (Figure 3B). The median OS was 18.9 months for patients receiving the first‐line combination therapy, and 13.4 months for patients experiencing second or later lines, with no statistically significant differences (*p* = 0.112) (Figure 3C), whereas PFS was significantly longer in patients treated in the first‐line setting than in patients treated in the second or later line setting (medians, 9.6 vs. 4.7 months; HR, 0.526; 95% CI, 0.329–0.841; *p* = 0.007) (Figure 3D). OS was significantly longer in patients with Child‐Pugh class A versus class B (medians, 18.8 vs. 5.9 months; HR, 0.235; 95% CI, 0.096–0.576; *p* = 0.002), as was PFS (medians, 9.1 vs. 4.4 months; HR, 0.381; 95% CI, 0.176–0.827; *p* = 0.015) (Figures 3E,F). Compared with patients with ALBI grade 2/3, those with ALBI grade 1 had significantly longer OS (medians, 23.5 vs. 13.4 months; HR, 0.543; 95% CI, 0.374–0.790; *p* = 0.001) and PFS (medians, 10.1 vs. 6.6 months; HR, 0.748; 95% CI, 0.537–1.038; *p* = 0.082) (Figure 3G,H).

### 3.3 Variables associated with OS and PFS

In univariate analyses, variables including male sex, Eastern Cooperative Oncology Group performance status (ECOG PS) 2, α‐fetoprotein (AFP) >400 ng/mL, and protein induced by vitamin K absence or antagonist‐II (PIVKA‐II) ≥2000 mAU/mL were demonstrated as significantly associated with PFS (Table 3). In multivariate analyses, independent associations with PFS were shown for ECOG PS 2 (HR, 2.081; 95% CI, 1.083–3.997; *p* = 0.028) and PIVKA‐II ≥2000 mAU/mL (HR, 1.653; 95% CI, 1.168–2.340; *p* = 0.005) (Table 3). Moreover, significant univariate associations with OS were shown for macrovascular invasion, ECOG PS 2, maximum tumor diameter > 10 cm, AFP > 400 ng/mL, and PIVKA‐II ≥2000 mAU/mL. Among these, independent prognostic significance for OS was shown for ECOG PS 2 (HR, 3.220; 95% CI, 1.611–6.437; *p* = 0.001) and PIVKA‐II ≥2000 mAU/mL (HR, 2.057; 95% CI, 1.377–3.073; *p* = 0.001).

**TABLE 3:** Variables associated with PFS and OS in the overall cohort.

Variable	PFS				OS			
	Univariate		Multivariate		Univariate		Multivariate	
	HR (95% CI)	*p* value	HR (95% CI)	*p* value	HR (95% CI)	*p* value	HR (95% CI)	*p* value
Age >60 years	1.007 (0.718–1.414)	0.966			1.160 (0.796–1.691)	0.440		
Male sex	0.568 (0.341–0.945)	0.030			0.638 (0.370–1.100)	0.106		
Extrahepatic metastasis	1.204 (0.860–1.685)	0.281			1.198 (0.822–1.749)	0.348		
Macrovascular invasion	1.053 (0.756–1.465)	0.761			1.531 (1.053–2.224)	0.026		
ECOG PS 2	2.306 (1.205–4.416)	0.012	2.081 (1.083–3.997)	0.028	3.447 (1.727–6.877)	0.001	3.220 (1.611–6.437)	0.001
Maximum tumor diameter >10 cm	1.162 (0.834–1.620)	0.374			1.562 (1.071–2.277)	0.020		
AFP >400 ng/mL	1.533 (1.097–2.143)	0.012			1.887 (1.288–2.764)	0.001		
HBsAg positivity	1.450 (0.934–2.251)	0.098			1.159 (0.714–1.880)	0.552		
PIVKA‐II ≥2000 mAU/mL	1.701 (1.204–2.403)	0.003	1.653 (1.168–2.340)	0.005	2.099 (1.406–3.134)	0.001	2.057 (1.377–3.073)	0.001

Abbreviations: AFP, α‐fetoprotein; ALBI, albumin‐bilirubin; BCLC, Barcelona Clinic Liver Cancer; CA 19–9, carbohydrate antigen 19–9; CEA, carcinoembryonic antigen; CI, confidence interval; ECOG PS, Eastern Cooperative Oncology Group performance status; HBsAg, hepatitis B surface antigen; HR, hazard ratio; OS, overall survival; PFS, progression‐free survival; PIVKA‐II, protein induced by vitamin K absence or antagonist‐II.

### 3.4 Safety outcomes

Grade 3/4 AEs were managed with dose reduction/interruption/discontinuation unless transient during the treatment interval, as recommended by American Society of Clinical Oncology treatment guidelines or relative expert consensus.^26^, ^27^ The dose of the anti‐PD‐1 antibody was reduced in 25 patients (11.9%), while the dose of lenvatinib was reduced in 37 patients (17.6%). Treatment with lenvatinib or anti‐PD‐1 antibodies was interrupted in 20 patients (9.5%) due to AEs. AEs that led to discontinuation of combination treatment included increased blood bilirubin in 11 patients (5.2%), hypertension in 5 patients (2.4%), diarrhea in 5 patients (2.4%), and gastrointestinal bleeding in 3 patients (1.4%). As shown in Table 4, the most frequently reported treatment‐related AEs of any grade were increased blood bilirubin (42.4%), elevated alanine aminotransferase (ALT)/aspartate aminotransferase (AST) (39.5%), and diarrhea (28.1%), while the most common grade 3/4 AEs were elevated ALT/AST (28.1%), increased blood bilirubin (23.3%), diarrhea (13.8%), and hand‐foot skin reaction (9.0%).

**TABLE 4:** Frequency of AEs of any grade or grade 3/4 in patients with ALBI grade 1 versus 2/3.

Patients, *n* (%)	AEs of any grade			Grade 3/4 AEs		
	ALBI grade 1	ALBI grade 2/3	*p* value	ALBI grade 1	ALBI grade 2/3	*p* value
	(*n* = 104)	(*n* = 106)		(*n* = 104)	(*n* = 106)	
Any adverse events	79 (76.0)	88 (83.0)	0.205	40 (38.5)	61 (57.5)	0.006
Hypertension	25 (24.0)	24 (22.6)	0.811	10 (9.6)	13 (12.3)	0.539
Skin rash	8 (7.7)	6 (5.7)	0.555	4 (3.8)	2 (1.9)	0.443
Hand‐foot skin reaction	18 (17.3)	31 (29.2)	0.041	8 (7.7)	11 (10.4)	0.498
Diarrhea	30 (28.8)	29 (27.4)	0.810	12 (11.5)	17 (16.0)	0.345
Fatigue	14 (13.5)	25 (23.6)	0.059	6 (5.8)	18 (17.0)	0.016
Increased blood bilirubin	38 (36.5)	51 (48.1)	0.090	15 (14.4)	34 (32.1)	0.002
Elevated AST/ALT	34 (32.7)	49 (46.2)	0.045	20 (19.2)	39 (36.8)	0.005
Thrombocytopenia	17 (16.3)	12 (11.3)	0.318	6 (5.8)	8 (7.5)	0.606
Decreased appetite	19 (18.3)	32 (30.2)	0.044	10 (9.6)	19 (17.9)	0.109
Hypothyroidism	6 (5.8)	2 (1.9)	0.168	4 (3.8)	1 (0.9)	0.210
Pneumonitis	1 (1.0)	4 (3.8)	0.369	1 (1.0)	4 (3.8)	0.369
Hemorrhage	4 (3.8)	2 (1.9)	0.443	4 (3.8)	2 (1.9)	0.443

Abbreviations: AE, adverse event; ALBI, albumin‐bilirubin; ALT, alanine aminotransferase; AST, aspartate aminotransferase.

Patients with ALBI grade 2/3 had a similar rate of AEs of any grade to patients with ALBI grade 1 (83.0% vs. 76.0%, respectively; *p* = 0.205), but a higher rate of grade 3/4 AEs (57.5% vs. 38.6%; *p* = 0.006) (Table 4). Furthermore, the following grade 3/4 AEs were significantly more common in patients with ALBI grade 2/3 than in those with ALBI grade 1: increased bilirubin (32.1% vs. 14.4%, *p* = 0.002); elevated ALT/AST (36.8% vs. 19.2%; *p* = 0.005); and fatigue (17.0% vs. 5.8%; *p* = 0.016). In patients with Child‐Pugh class A and B, there were no significant differences in the rates of AEs of any grade (79.5% vs. 80.0%, *p* = 0.962) or grade 3/4 (46.7% vs. 66.7%, *p* = 0.135) (Table S3).

## 4 DISCUSSION

The current treatment landscape for advanced HCC comprises both targeted agents and immunotherapies, but the optimal approach for combining and/or sequencing these agents remains to be defined. Among the various regimens evaluated in the clinical trials to date, the highest ORR was achieved with lenvatinib plus PD‐1 antibodies.^9^, ^10^ Furthermore, the remarkable efficacy of targeted therapy plus ICB combinations has, in some cases, downstaged the tumor sufficiently to enable patients with initially inoperable tumors to undergo resection.^23^

In real‐world cohorts, median OS with monotherapy of lenvatinib was reported at approximately 10–15 months, with ORRs of 42.1%, 22.2%, and 18.9% in Japan,^28^ China,^29^ and Korea,^30^ respectively, while a European study of PD‐1‐targeted ICBs reported a median OS of 11 months and ORR of 12.3%.^31^ Recently, a real‐world study of combination therapy with tyrosine kinase inhibitors plus anti‐PD‐1 antibodies reported an ORR per RECIST v1.1 of 36.7%.^32^ Zhu et al. reported a cohort of 72 patients receiving lenvatinib‐based combination therapy reaching median OS of 99 weeks with ORR of 26.4%.^33^ In the studies including second or later line treatment, the median OS of patients receiving second or later line treatment decreased to varying degrees. Maruta et al. reported the median OS of 5.2 months in the first‐line treatment cohort, and 4.8 months in second or later line patients who received lenvatinib for the expanded indication from the REFLECT trial.^34^ In another Korean study, the median OS of first‐line lenvatinib treatment was 10.7 months, while that of second or later line lenvatinib treatment was 6.4 months.^35^ Although lower than the results of IMbrave150^8^ and LEAP‐002,^12^ the median OS of the overall cohort in this study of lenvatinib plus anti‐PD‐1 antibodies was higher than the data reported in previous clinical trials or real‐world studies of monotherapy, proving the effectiveness of combination treatment to some extent. Besides, the present study enrolled 81.9% of patients received first‐line treatment and showed a median OS of 18.9 months, a result comparable to that of LEAP‐002 trial. The median OS of second or later line treatment is considerable with 1‐year survival rate over 50%. Moreover, the OS and ORR did not differ significantly between second or later line treatment and first‐line treatment, largely due to the small number of patients in the second or later line setting. The above results are exciting and demonstrate the benefit of combination therapy for patients with advanced or unresectable advanced liver cancer, encouraging the application of the treatment regimen in clinical practice, and the larger cohort in this study presented results similar to some previous small sample studies, demonstrating the generalizability of the conclusions.

Due to the fundamental difference in inclusion criteria to clinical trials, the study is likely to yield results impacted by the inclusion patient subsets with poor baseline liver function. Liver function parameters were initially studied in HCC as predictors of postoperative liver failure, and are increasingly gaining relevance in patients undergoing nonsurgical treatment. A retrospective study of lenvatinib reported shorter OS and progression‐free survival in patients with ALBI grade 2b/3 than those with ALBI grade 1/2a.^36^ Another study evaluating the effectiveness of nivolumab demonstrated poorer ORR and OS in patients with Child‐Pugh class B versus class A.^37^ Similarly, in the present study, reduced OS were associated with poor baseline liver function, whether defined according to Child‐Pugh class or ALBI grade. In contrast, both ORR and DCR did not differ significantly between liver function subgroups. These observations are consistent with an effect of poor liver function in decreasing drug tolerability, leading to premature treatment interruption or discontinuation, rather than directly reducing anti‐tumor efficacy. To some extent, the results mentioned above recommend the use of combination therapy of lenvatinib with anti‐PD‐1 antibodies in patients with sufficient liver function reserve in clinical practice, while discouraging patients with Child‐Pugh B class and ALBI grade 2/3 from the combination treatment.

Most clinical trials on liver cancer require patients with ECOG PS 0–1,^38^ with several studies suggesting the association between better therapeutic effects for liver cancer and ECOG PS 0–1.^39^ In the present study, ECOG PS 2 was demonstrated to be associated with worse OS and PFS, as patients with ECOG PS 2 are probably less tolerant to combination therapies, suggesting monotherapy or other agents with fewer toxic effects as better choices for this subset of patients. PIVKA‐II was considered useful for the treatment evaluation of HCC, in pre‐treatment evaluation to screen the appropriate treatment population,^40^ and in post‐treatment evaluation of treatment response.^41^ Our results also demonstrated poor prognostic in terms of OS of baseline PIVKA‐II in patients treated with lenvatinib plus anti‐PD‐1 antibody therapy.

Treatment‐related AEs with these combination therapy regimens reflect the established safety profiles of the constituent targeted therapy and ICB agents. In the present study, the incidence of treatment‐related AEs was overall manageable and generally in line with expectations. Indeed, higher rates of AEs were previously reported with lenvatinib plus pembrolizumab in KEYNOTE‐524 (treatment‐related AE rates: 95% for any grade and 67% for grade ≥3) and with camrelizumab plus apatinib in the RESCUE trial (treatment‐related AE rates: 99% for any grade and 77% for grade ≥3)^9^, ^11^ than in our study (treatment‐related AE rates: 79.5% for any grade and 48.1% for grade ≥3). Moreover, ALBI grade 2/3 was associated with a higher incidence of elevated AST/ALT, hand‐foot skin reaction and decreased appetite of any grade, as well as grade 3/4 AEs overall in our real‐world cohort. The higher incidence of grade 3/4 AEs may contribute to the shorter duration of treatment, leading to shorter OS in patients with poorer liver function in clinical practice. The lack of significant differences between patients with Child‐Pugh class A and B in the overall rates of AEs of any grade or grade 3/4 likely results from the few number of patients with Child‐Pugh class B included in the study.

The strength of our study is the largest cohort using lenvatinib plus anti‐PD‐1 antibodies under conditions of real‐life clinical practice to date, mainly consisted of BCLC stage C HCC and more than half of patients with ALBI grade 2/3. The main limitations of this study include the retrospective, single‐center design and the inclusion of relatively few individuals with Child‐Pugh class B, which limits the generalizability of the conclusion. Although several clinical experiments and our previous findings suggest comparable efficacy of varied PD‐1 therapies/doses,^20^, ^21^, ^22^ this may still carry potential differences in treatment outcomes, AEs, etc.

## 5 CONCLUSION

Combination therapy with lenvatinib plus anti‐PD‐1 antibody showed potent anti‐tumor efficacy in patients with sufficient liver function reserve, and further study is needed for those with Child‐Pugh B class and ALBI grade 2–3, for the sake of improved efficacy and reduced AEs. The present study was performed on the largest cohort of combination therapy for treatment of advanced HCC under conditions of real‐life clinical practice to date.

## AUTHOR CONTRIBUTIONS

**Ming‐Hao Xu:** Conceptualization (equal); data curation (equal); formal analysis (equal); writing – original draft (equal). **Cheng Huang:** Data curation (equal). **Mei‐Ling Li:** Data curation (equal). **Xiao‐Dong zhu:** Data curation (equal); formal analysis (equal). **Chang‐Jun Tan:** Data curation (equal). **Jian Zhou:** Data curation (equal). **Jia Fan:** Data curation (equal). **Hui‐Chuan Sun:** Formal analysis (equal); funding acquisition (equal); writing – review and editing (equal). **Ying‐Hao Shen:** Conceptualization (lead); funding acquisition (equal); writing – review and editing (lead).

## FUNDING INFORMATION

National Natural Science Foundation of China (NSFC) (Grant No. 82172799, 81672326, and 81871929).

## CONFLICT OF INTEREST STATEMENT

No conflicts of interest were declared.

## STATEMENT OF ETHICS AND STUDY APPROVAL AND CONSENT TO PARTICIPATE

The study was approved by the Zhongshan Hospital Research Ethics Committee. Written informed consent was signed by patients before combination treatment.

## Supporting information


Table S1.
Table S2.
Table S3.

## Data Availability

The raw data supporting the conclusions of this article will be made available by the corresponding author, without undue reservation.
