# Supplementary material for: Effectiveness and safety of lenvatinib plus anti‐programmed death‐1 antibodies in patients with hepatocellular carcinoma: A real‐world cohort study
Source: Cancer Med. 2023 Feb 15;12(8):9202–12. doi: 10.1002/cam4.5661 (PMC10166966; doi:10.1002/cam4.5661)
Supplement: Supplementary file 1 — Table S1. Table S2. Table S3. [file CAM4-12-9202-s001.docx]

**Supplemental Table 1. Anti-PD-1 antibody use in the overall cohort**

| Anti-PD-1 antibody | Patients, n (%) |
| --- | --- |
| Sintilimab (Innovent Biologics, China) | 78 (37.1) |
| Camrelizumab (Hengrui Medicine, China) | 65 (31.0) |
| Pembrolizumab (MSD, USA) | 24 (11.4) |
| Toripalimab (Junshi Bioscience, China) | 17 (8.1) |
| Tislelizumab (Novartis, China) | 15 (7.1) |
| Nivolumab (Bristol-Myers Squibb, USA) | 11 (5.2) |

PD-1, programmed death-1.

**Supplemental Table 2. Prior treatments of second-line combination treatment patients.**

| Prior Treatment | Patients, n (%) |
| --- | --- |
| Sorafenib | 10 (28.6) |
| Sorafenib plus TACE | 9 (25.7) |
| Regorafenib | 6 (17.1) |
| Apatinib | 6 (17.1) |
| Apatinib plus TACE | 4 (11.4) |

TACE, transcatheter arterial chemoembolization.

**Supplemental Table 3. Frequency of AEs of any grade or grade 3/4 in patients with Child–Pugh class A vs B**

| Patients, n (%) | AEs of any grade | | | Grade 3/4 AEs | | |
| --- | --- | --- | --- | --- | --- | --- |
|  | **Child-Pugh A (n = 195)** | **Child-Pugh B (n = 15)** | ***p* value** | **Child-Pugh A (n = 195)** | **Child-Pugh B (n = 15)** | ***p* value** |
| Any AE | 155 (79.5) | 12 (80.0) | 0.962 | 91 (46.7) | 10 (66.7) | 0.135 |
| Hypertension | 47 (24.1) | 2 (13.3) | 0.342 | 22 (11.3) | 1 (6.7) | 0.581 |
| Skin rash | 13 (6.7) | 1 (6.7) | 1.000 | 5 (2.6) | 1 (6.7) | 0.358 |
| Hand-foot skin reaction | 46 (23.6) | 3 (20.0) | 0.751 | 18 (9.2) | 1 (6.7) | 0.739 |
| Diarrhea | 57 (29.2) | 2 (13.3) | 0.187 | 28 (14.4) | 1 (6.7) | 0.405 |
| Fatigue | 34 (17.4) | 5 (66.7) | 0.127 | 21 (10.8) | 3 (20.0) | 0.279 |
| Increased blood bilirubin | 83 (42.6) | 6 (40.0) | 0.846 | 45 (23.1) | 4 (26.7) | 0.751 |
| Elevated AST/ALT | 78 (40.0) | 5 (33.3) | 0.611 | 57 (29.2) | 2 (13.3) | 0.187 |
| Thrombocytopenia | 29 (14.9) | 0 (0) | 0.108 | 14 (7.2) | 0 (0) | 0.283 |
| Decreased appetite | 47 (24.1) | 4 (26.7) | 0.823 | 29 (14.9) | 0 (0) | 0.108 |
| Hypothyroidism | 7 (3.6) | 1 (6.7) | 0.549 | 5 (2.6) | 0 (0) | 0.530 |
| Pneumonitis | 5 (2.6) | 0 (0) | 0.530 | 5 (2.6) | 0 (0) | 0.530 |
| Hemorrhage | 4 (2.1) | 2 (13.3) | 0.011 | 4 (2.1) | 2 (13.3) | 0.011 |

AE, adverse event; ALT, alanine aminotransferase; AST, aspartate aminotransferase.
